# Lifetime leisure music exposure associated with increased frequency of tinnitus

**DOI:** 10.1016/j.heares.2016.10.030

**Published:** 2017-04

**Authors:** David R. Moore, Oliver Zobay, Robert C. Mackinnon, William M. Whitmer, Michael A. Akeroyd

**Affiliations:** a MRC Institute of Hearing Research, University Park, Nottingham, NG7 2RD, UK; b Communication Sciences Research Center, Cincinnati Children Hospital Medical Center, Cincinnati, OH, 45229, USA; c Department of Otolaryngology, University of Cincinnati College of Medicine, Cincinnati, OH, 45229, USA; d School of Medicine, University of Nottingham, Nottingham, NG7 2UH, UK; e MRC/CSO Institute of Hearing Research - Scottish Section, Glasgow Royal Infirmary, Glasgow, G31 2ER, UK

**Keywords:** Personal music players, Speech-in-noise, Hearing difficulty, Internet, Hearing aids, OR, odds ratio, SRT, speech reception threshold, MRC, Medical Research Council, Hf-DTT, High Frequency Digit Triplets Test, PMP, personal music player, AIC, Akaike Information Criterion, CI, confidence interval

## Abstract

Tinnitus has been linked to noise exposure, a common form of which is listening to music as a leisure activity. The relationship between tinnitus and type and duration of music exposure is not well understood. We conducted an internet-based population study that asked participants questions about lifetime music exposure and hearing, and included a hearing test involving speech intelligibility in noise, the High Frequency Digit Triplets Test. 4950 people aged 17–75 years completed all questions and the hearing test. Results were analyzed using multinomial regression models. High exposure to leisure music, hearing difficulty, increasing age and workplace noise exposure were independently associated with increased tinnitus. Three forms of music exposure (pubs/clubs, concerts, personal music players) did not differ in their relationship to tinnitus. More males than females reported tinnitus. The objective measure of speech reception threshold had only a minimal relationship with tinnitus. Self-reported hearing difficulty was more strongly associated with tinnitus, but 76% of people reporting usual or constant tinnitus also reported little or no hearing difficulty. Overall, around 40% of participants of all ages reported never experiencing tinnitus, while 29% reported sometimes, usually or constantly experiencing tinnitus that lasted more than 5 min. Together, the results suggest that tinnitus is much more common than hearing loss, but that there is little association between the two, especially among the younger adults disproportionately sampled in this study.

## Highlights

• Internet study of lifetime music exposure and tinnitus sampled 4950 people, 17–75 years old.
• High music exposure, hearing difficulty, increasing age associated with increased tinnitus.
• 40% of people of all ages reported no tinnitus; 29% reported at least some tinnitus.
• Little association was found between tinnitus and hearing loss.

## 1 Introduction

Noise exposure has been linked to hearing loss and tinnitus, most recently in the high rate of both hearing disorders among military personnel exposed to gunfire and explosions (Elgoyhen et al., 2015, Helfer, 2011). However, a much more common source of noise exposure is that produced by listening to music as a leisure activity. Most reports of the potentially damaging effects on hearing of listening to music focus on hearing loss (Buckey et al., 2015, Halevi-Katz et al., 2015, Helleman and Dreschler, 2015, Opperman et al., 2006) but, as shown for the military, tinnitus may cause equal or greater life-limiting disability (Elgoyhen et al., 2015, Langguth et al., 2013, McCormack et al., 2015, Wunderlich et al., 2015). Epidemiological studies of tinnitus have generally not characterized in detail the source of noise exposure, for example grouping all forms of leisure exposure into a single yes/no question (Shargorodsky et al., 2010). One recent, large study of people aged 40–69 years that did separate ‘loud music’ exposure from other sources of noise (McCormack et al., 2014b) found the puzzling result that ‘transient’ and ‘persistent’ tinnitus were significantly (p < 0.001) associated with music exposure (odds ratio, OR = 1.51 and 1.56 respectively, relative to ‘no’ tinnitus), but that ‘bothersome’ tinnitus was not significantly associated (OR = 1.07; p = 0.18). In the study reported here we examined the frequency of tinnitus as a function of age and estimated lifetime music exposure as part of a large-scale, internet-based public science experiment on hearing.

Tinnitus is highly prevalent in the population. Based on self-report in a large, middle-aged UK population, current tinnitus rose from 12% of 40–44 year olds (y.o.) to 24% of 65–69 y.o. (Dawes et al., 2014). Tinnitus was more prevalent among men (18%) than women (14%) at all ages. However, the proportion of those with current tinnitus who reported their tinnitus to be ‘bothersome’ was greater for women (25%) than for men (22%; (McCormack et al., 2014b). Tinnitus has also been independently related to hearing loss, whether hearing is measured using pure tone, audiometric threshold (Langguth et al., 2013, Shargorodsky et al., 2010), or threshold identification of speech sounds in the presence of background noise (speech reception threshold, SRT; (McCormack et al., 2014a). In their study (McCormack et al., 2014b), classified SRTs as ‘normal’, ‘insufficent’ or ‘poor’ (see (Dawes et al., 2014) and found that, relative to those with normal hearing, participants with insufficient or poor hearing had significantly (p < 0.001) more ‘transient’, ‘persistent’ and ‘bothersome’ tinnitus. However, as above, participants with ‘bothersome’ tinnitus had smaller ORs than those with persistent tinnitus.

The present study was conducted as part of a large, public-participation experiment to celebrate the Centenary in 2013 of the UK Medical Research Council (MRC). Participants were invited to visit a purpose-designed website where they answered questions about their music exposure and hearing, including a question about the frequency of tinnitus. They also completed a hearing test involving speech intelligibility in noise. Questions can be sensitive indicators of environmental history (Chavarro et al., 2009). The High Frequency Digit Triplets Test (Hf-DTT), used here, asks listeners to identify sets (trials) of three successive monosyllabic digits presented against a speech-shaped noise masker. The masker was low-pass filtered, making the test sensitive to higher frequency hearing loss that has been associated with noise exposure.

The aims of this research were to determine the relation between tinnitus frequency (rate of occurrence) and lifetime leisure music exposure across age and other variables that have been related to tinnitus, and to make this evidence visible to the public to inform impact of leisure music on tinnitus.

## 2 Materials and methods

The website used for this study, including the hearing test, is still available although data collection is complete. The site (http://www.100yearsofamplifiedmusic.org/#) provided information about hearing and the history of amplified music, asked questions about current hearing and lifetime music exposure, and delivered the Hf-DTT. Data were collected without personal identifiers.

### 2.1 Participants

Participation was solicited by direct email among various employee groups, notably the (then) current staff and students of the MRC and of Cincinnati Children's Hospital. In addition, MRC organized various public events and communications, including lectures, radio interviews and newspaper reports in which the study and website were publicised. Participation was dependent on use of a computer connected to the internet. Preliminary questions asked for year of birth and gender.

**Fig. 1:**
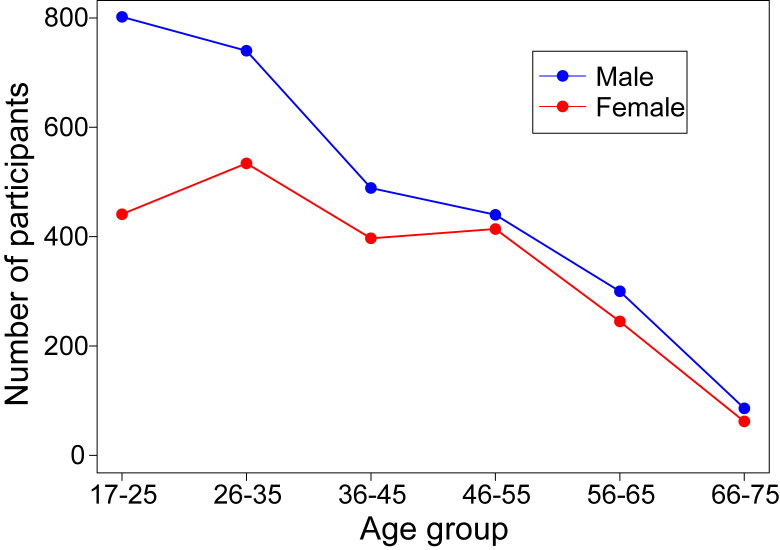
Number of participants by age group and gender.

5298 people completed all the questions and the hearing test. People younger than 17 years (n = 208) were excluded by participation criteria. Those older than 75 years (n = 16 aged 76–80 years, n = 15 older than 80) were also excluded due to heterogeneity by age and smallness in number. Data from users of hearing aids (aged 17–75 years; n = 106) were examined separately, but excluded from the main analysis, along with that of cochlear implant users (n = 3), leaving a total sample of 4950. The age and gender distribution of the participants is shown in Fig. 1. The sample was biased strongly in favour of younger and male participants.

### 2.2 Questions

The question concerning tinnitus was “How often nowadays do you get tinnitus (noises such as ringing or buzzing in your heard or ears) that lasts for more than 5 min?” with response options: Never, Rarely, Sometimes, Usually, Constantly (Appendix B, Question 5).

The full list of questions concerning leisure music experience, and their response options, is in Appendix A. Questions and responses concerning hearing, tinnitus and other noise exposure are in Appendix B. Participants rated the frequency of music listening along a seven point ordinal scale for each of three listening environments, namely Concerts (including ‘gigs’ and festivals), Clubs (including ‘pubs’), and personal music players (PMPs; e.g. mobile phones, other MP3 players, personal CD or cassette players). For each environment, participants were asked to rate experience in each decade of their life (10–19, 20–29,. . . 70–79, 80+) up to and including the decade of their present age.

### 2.3 High frequency digit triplets test

The development, validation and normalization of the English HF-DTT in normal hearing and hearing impaired listeners is described in detail elsewhere (Vlaming et al., 2014). Briefly, it is a measure of speech-in-noise hearing that emphasizes high frequency hearing by presenting speech stimuli (the digits 0–9) against a quasi-stationary, speech-shaped masking noise that is digitally filtered to provide about 10–15 dB decreased masking in the high frequency range (>1500 Hz), relative to the masker used original Dutch DTT (Smits et al., 2004). The online Hf-DTT is robust to the use of different levels and speakers primarily because the test itself does not depend on calibrated equipment and SRT remains relatively constant over a wide dynamic range, including the range that most people find comfortable (Vlaming et al., 2014). It has also been shown in previous research that reliable data can be obtained using online delivered DTT testing (Stam et al., 2015). Groups of three digits (e.g. 5-7-2), preceded by a trailer, “The digits …” were presented in each of 25 trials. Listening was binaural (diotic) via the listener's chosen mode of delivery (headphones, computer speakers etc.; see Appendix B). Prior to the first trial, the listener was asked to adjust the overall level of signal + noise to a “comfortable level”. On each trial the noise level was held at this level and the signal level was varied adaptively dependent on the listener correctly identifying all three digits. The SRT was the mean speech-masker ratio averaged over the last 19 trials.Table 1.

### 2.4 Analysis

A key technical issue of this study concerned the best way to enter the music exposure variables (Decade of life; Type of exposure: Clubs, Concerts, PMPs) into the statistical analyses. There are many possibilities of how this can be done, for example, as a set of decade-wise scores or as a single aggregate score such as the total or mean lifetime exposure. Using the Akaike Information Criterion (AIC), a method based on information theory to assess the quality of different statistical models (Bozdogan, 1987), several possibilities were explored. These included (a) means and total scores after transforming response categories into a 1–7 Likert-type scale (1–5 for PMP), (b) yearly means and lifetime totals of club or concert visits, estimated from the response categories, and (c) logarithmic transforms of the quantities in (b). Note that transformations of type (b) and (c) could not be defined for PMP so, for comparability reasons, scores were computed using the same algorithms as for Clubs and Concerts. These transforms, (a)-(c), were compared across Clubs, Concerts and PMP. Overall results were robust and consistent. For example, we estimated the models presented in Table 2, Table 5 using each transformation. For Table 2, the range of odds and ORs was generally comparable with or smaller than the width of the confidence intervals. Across all parameters, the average range was 0.08 with a maximum of 0.22. Analogous conclusions were obtained from the analyses for Table 5. We therefore chose to use the mean Likert score, type (a), hereafter the ‘mean music exposure’, as this measure is simple to explain and compute.

#### 2.4.1 Descriptive analysis of effects of music exposure

Participants were divided into 10-year age groups (except a young, 17–25 y.o. group) and within each age group a median split, separating participants into low and high exposure groups was performed based on the mean music exposure. Tinnitus prevalence was computed within each subgroup defined by age-band and degree (low/high) of music exposure.

#### 2.4.2 Formal statistical analysis

All statistical analyses were conducted with R software. The relationship between tinnitus prevalence and potential explanatory variables was investigated with the help of multinomial logit regression models (Agresti, 2013) using the mlogit and nnet packages running under R. In this type of model the ORs of the different levels of each variable (e.g. ‘some’ and ‘constant’ tinnitus) relative to a base level (e.g. ‘no’ tinnitus) are described by a separate set of coefficients. These models were found to reproduce the observed behavior of the tinnitus prevalence better than less flexible ordinal regression models.

As potential explanatory variables, our regression models included (i) age, gender, workplace noise, mean music exposure (Appendix A), (ii) the first-order interaction between these variables, and (iii) the quadratic effects of age and music exposure. Model selection based on likelihood ratio tests and AIC was performed to find the model that offered the best compromise between parsimony and data fit. Subjective hearing assessments and SRT were not included in the model as they, together with tinnitus, could be interrelated indicators of an overall, underlying (latent) hearing status. Statistically, including these variables might therefore distort the estimates for the associations of the explanatory variables. Additional models were examined that did not include ‘music’ and ‘noise’, but did include ‘recent noise’ (Appendix B) in order to isolate that variable.

In separate analyses, the relation between tinnitus and SRT was examined using multiple linear regression models, with SRT as dependent variable. Starting from a comprehensive model containing (a) tinnitus, (b) age, gender, music, noise, recent noise and sound delivery system as further potential explanatory variables, and (c) all two-way interactions, we applied backwards regression based on the AIC criterion to select a well-fitting simpler model.

#### 2.4.3 Illustration of predicted effect size

To translate the results of the statistical modelling into an easily interpretable quantitative measure of the predicted effect size of music exposure, we first computed the means of the lifetime-average music exposure for the low- and high-exposure groups in each of the age bands, as defined by a median split in each band. These means provided a measure of the typical age-dependent spread of exposure across participants. Next, for these means, and the corresponding age, we obtained the respective predictions of a statistical model in which we regressed Tinnitus against Age and Music exposure in first and second order (in concise symbolic R notation: Tinnitus ∼ Age + Music + Musicˆ2) and plotted them together with the observed tinnitus prevalence. A similar approach was used to illustrate the age-dependent effects of workplace noise and hearing aids on tinnitus, and of tinnitus on SRT. In these cases, the sample subgroups were defined by the level of the independent factor.

## 3 Results

**Fig. 2:**
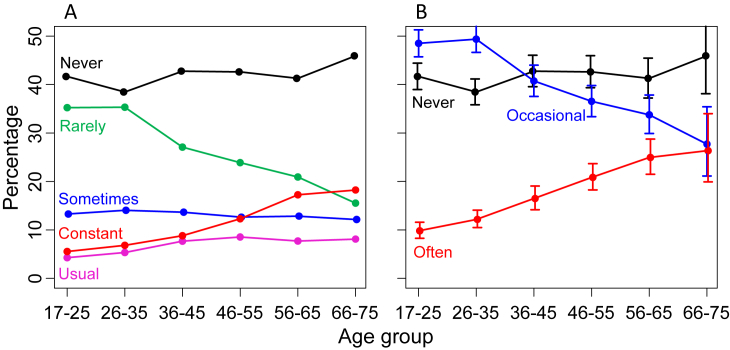
Prevalence of tinnitus by age. **A**. Percent responses to the 5 options posed in the question about tinnitus. **B**. Percent responses regrouped to a simpler, 3-option grouping as elaborated in text.

59% of the sample reported having tinnitus that lasted more than 5 min at least some of the time (Fig. 2). The overall prevalence of tinnitus increased gradually with age, largely accounted for by an increase in those reporting ‘Constant’ tinnitus and a decrease in reports of ‘Rare’ tinnitus (Fig. 2A). Surprisingly, the proportion of participants ‘Never’ experiencing tinnitus remained relatively constant across age. For increased power and simplicity in further analysis, three levels of tinnitus were distinguished, ‘Never’, ‘Occasional’ (responding ‘Rarely’ + ‘Sometimes’), and ‘Often’ (responding ‘Usual’ + ‘Constant’). Prevalence of these three levels is shown with 95% confidence intervals in Fig. 2B.

### 3.1 Music exposure

Table 2 provides a summary of the final multinomial regression model for tinnitus prevalence. The model contains age, gender, age-gender interaction, workplace noise, and lifetime music exposure (linear and quadratic terms) as predictive variables. Table 2A shows ‘odds’ and ORs for the tinnitus question outcomes ‘Occasional’ and ‘Often’ relative to ‘Never’. Odds <1 indicate a reduced probability for a ‘baseline’ participant having ‘Often’ or ‘Occasional’ tinnitus compared to ‘Never’ having tinnitus. ORs greater/less than 1 indicate increased/decreased odds of tinnitus relative to baseline for the specified model term. Further details and examples are provided in Table 2A caption and in Appendix C.

**Fig. 3:**
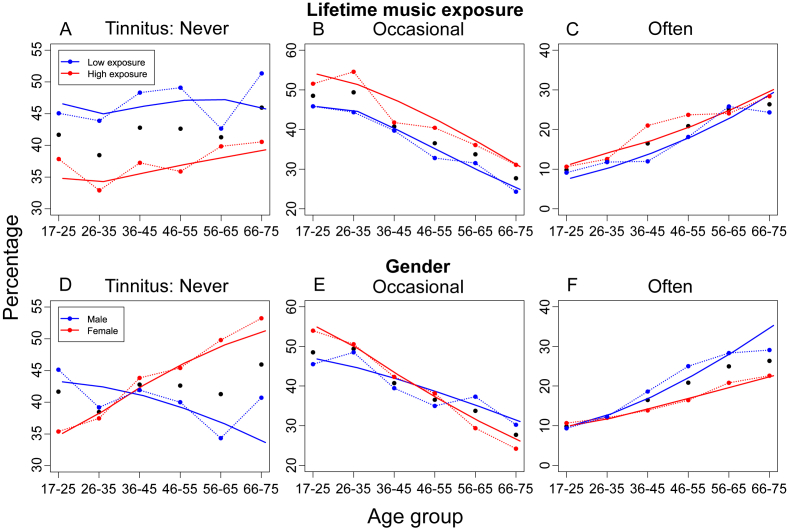
Music exposure and gender relation to tinnitus prevalence. Percentage respondents in each age group reporting each tinnitus frequency (black points; as in Fig. 2B). **A-C**. Relation to lifetime music exposure, divided into ‘Low’ and ‘High’. **D-F**. Relation to gender. For each figure, coloured points show mean data and lines show predictions from simplified multinomial regression models (Tinnitus ∼ Age + Music + Musicˆ2 and Tinnitus ∼ Age*Gender, respectively).

**Fig. 4:**
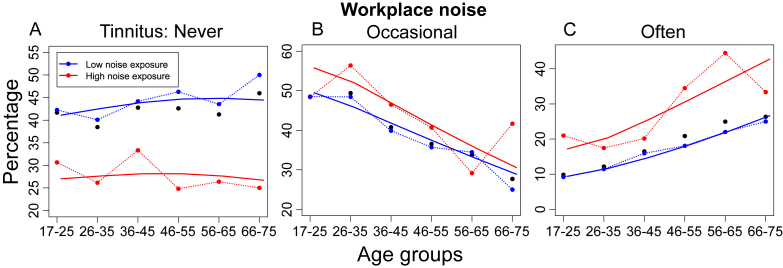
Workplace noise exposure and tinnitus prevalence. Details as per Fig. 3. Model: Tinnitus ∼ Age + Noise.

Key results are that the odds of having tinnitus occasionally or often increased with music exposure and workplace noise, and with increasing male age, but decreasing female age. These trends are also seen in Fig. 3, Fig. 4, which show observed age-dependent prevalence stratified according to music exposure, gender and workplace noise. The analysis of Deviance (Table 2B (Davison, 2008); showed that all terms retained in the model were significant (chi-square), including age, noise and music exposure, and age:gender interaction (all p < 0.001).

Fig. 3A–C shows that high music exposure reduced the likelihood of a ‘Never’ response by about 10 percentage points (pp) and correspondingly increased the likelihood of ‘Occasional’ and ‘Often’ responses. However, the impact of high lifetime music exposure on tinnitus frequency remained relatively constant across age. Prolonged workplace noise exposure produced a very similar, age-independent pattern of changes to tinnitus frequency (Fig. 4). In contrast, the effect of gender on tinnitus frequency interacted powerfully with age (Fig. 3D–F). In younger participants, male reports of ‘Never’ experiencing tinnitus increased by about 10 pp compared to females. For older males, however, male reports were about 15 pp lower than females. Presumably, the combination of these opposing trends resulted in the relatively unchanging, with age, whole-sample response of ‘Never’ (Fig. 2). Relative to females, responses of ‘Occasional’ and ‘Often’ became increasingly more common in older males (Fig. 3E and F), perhaps reflecting the higher prevalence of hearing loss in males with advancing age (Moore et al., 2014).

**Fig. 5:**
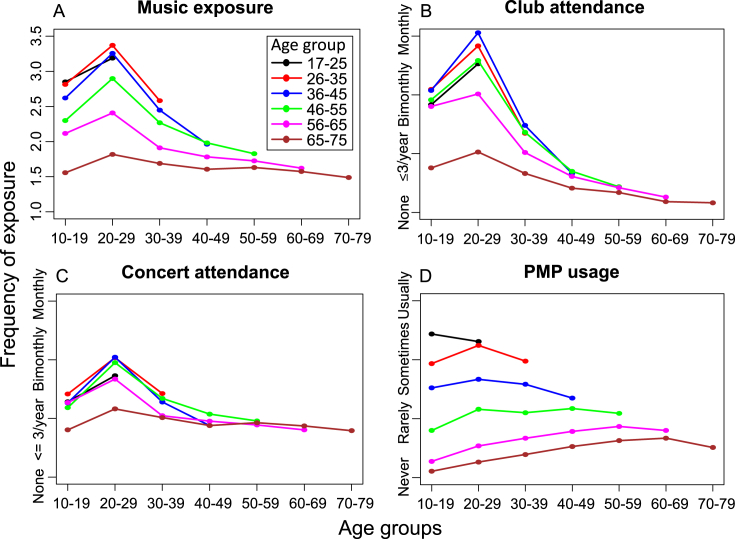
Relation between type and amount of music exposure by age decade. A. Total music exposure, and B-D. Each type of music exposure. Mean data points derived as per Methods.

Different forms of music showed similar exposure at a given age, but decreasing exposure with increasing age (Fig. 5). The correlation between music forms was low (PMP – Concerts, Clubs) to moderate (Concerts-Clubs; Table 3). When overall music exposure in the model of tinnitus prevalence was replaced by any of the specific forms of music (Clubs, Concert, PMPs), significant main effects of exposure were still observed (Table 4). In fact, they each had a similar effect profile to the overall model (Table 2B), suggesting that no one form of exposure produced a different effect from the others. Analysis using AIC indicated that mean overall exposure provided a better description of tinnitus variability than each of the three individual forms. Exposure was slightly higher among adult males in all but the youngest group (Table 4; frequency data not shown).

### 3.2 Speech reception threshold

**Fig. 6:**
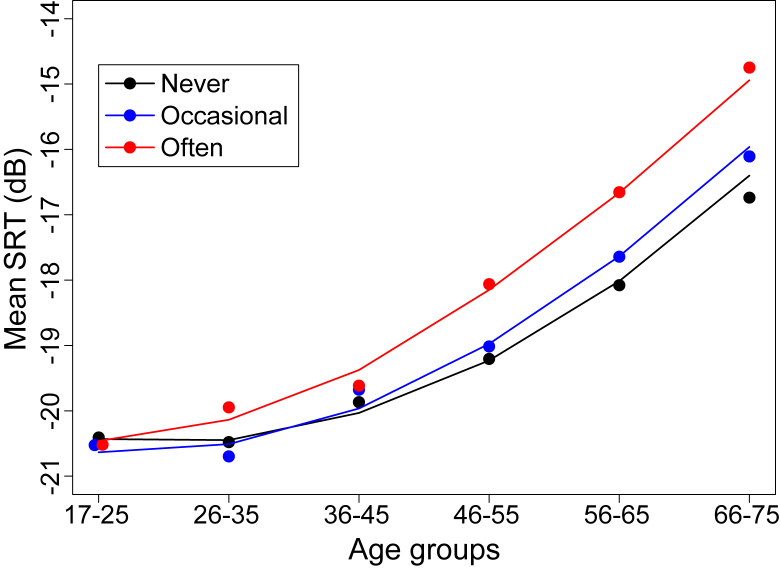
Relation between speech reception threshold (SRT) and tinnitus with age. Coloured points show mean data and lines show model predictions (SRT ∼ Age + Ageˆ2 + Tinnitus*Age).

SRT was related to age and tinnitus frequency (Fig. 6), as reported in other recent studies (McCormack et al., 2014b, Moore et al., 2014). We constructed multiple linear regression models of SRT that had tinnitus as an independent variable along with other potentially relevant predictors: age, gender, lifetime music exposure, workplace noise exposure, recent noise, sound delivery system, and their first-order interactions. The final model selected by a backwards regression procedure based on AIC is summarized in Table 5. Even after controlling for all confounding variables there is a remaining association between tinnitus and SRT (p < 0.001) that is modulated by the interaction with age, workplace noise and recent noise exposure. However, the correlation between tinnitus and SRT, using the original 5-level scale of the tinnitus questionnaire, was modest (r = 0.101, p < 0.001, CI: 0.074–0.129). After correcting for age, noise, and music, the partial correlation was just 0.061 (p < 0.001, CI: 0.033–0.089). In absolute terms, SRT was thus only minimally associated with tinnitus.

### 3.3 Hearing difficulty

Three questions were asked about hearing difficulty (Appendix B). As responses to the questions correlated well with one another (r = 0.61–0.69), we focus here on the general question “Do you currently have any difficulty with your hearing?” Table 6A shows the number of respondents with each frequency of tinnitus (5-level) against level of hearing difficulty. A clear relationship was found of increasing frequency of tinnitus with increasing hearing difficulty (r = 0.33, CI: 0.30–0.35, p < 0.001). That relationship persisted after controlling for Age, Noise, and Music (r = 0.29, CI: 0.27–0.32, p < 0.001). In modelling (Table 6B), tinnitus and age both explained considerably more variation in hearing difficulty, as measured by deviance, than workplace noise, music exposure and recent noise. But it is notable that most respondents with Usual or Constant tinnitus (76%) nevertheless reported no or only slight difficulty hearing. Each of the three questions concerning hearing difficulty correlated relatively weakly with SRT (r = 0.16–0.22).

### 3.4 Recent exposure to music or noise

A statistically significant effect of ‘recent exposure to loud music or loud noise’ on tinnitus was found in models that did not include workplace noise and lifetime music exposure (Table 7), but not in models that did include those two variables. The connection between recent exposure and tinnitus may be because recent exposure is correlated with mean lifetime music and noise exposure. Coding recent exposure levels of “None” as 0, “Music” and “Noise” as 1 each, and both “Noise and Music” as 2, the correlation with lifetime music exposure was 0.195. However, recent exposure did not explain any variability of longer-term tinnitus over and above that accounted for by lifetime music and workplace noise exposure.

### 3.5 Hearing aids

**Fig. 7:**
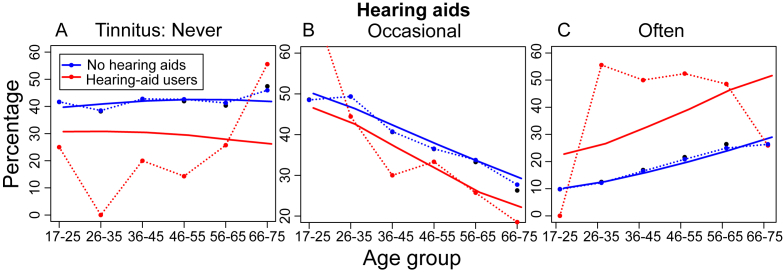
Hearing aid use and tinnitus prevalence. Details as per Fig. 3. Model: Tinnitus ∼ Age + Hearing Aid.

Hearing aid users were mostly (83/106) in the three oldest age groups. After allowing for the age bias in participant numbers, the prevalence of tinnitus was much higher among hearing aid users than among those who didn't use hearing aids (Fig. 7). Modeling is imprecise with such small numbers, but suggested an age-related 15–25 pp increase in prevalence of tinnitus ‘Often’ (3-level scale) among hearing aid users (Fig. 7C).

**4. Discussion**The data from this public-science experiment demonstrated that both increasing age and high exposure to leisure music increased the percentage of people reporting tinnitus. However, these trends occurred in parallel and almost independently of each other, suggesting a dissociation between age and the effect of leisure music on tinnitus. The three forms of music exposure did not differ significantly in terms of their relationship to tinnitus. Another parallel result was found of increasingly persistent tinnitus with duration of workplace noise exposure. However, the interaction between age and workplace noise was not statistically significant. More males than females reported tinnitus, and here there was a significant interaction with age; older males were more likely to report tinnitus ‘Often’ whereas, in the youngest groups, more females reported at least ‘Occasional’ tinnitus. Tinnitus frequency increased with increasing (i.e. poorer) SRT, but the relationship was weak compared with the influence of age on SRT. Finally, there was an association between tinnitus and perceived hearing difficulty that was not accounted for by the other explanatory variables. Overall, subjective reports of music exposure, noise exposure and hearing difficulty were all clearly associated with tinnitus frequency, whereas the objective measure of SRT had only a minimal relationship with tinnitus when adjusted for age.

### 3.6 Forms and duration of music exposure

This is the first study of which we are aware that has examined different forms of leisure music exposure in relation to tinnitus and to have quantified music exposure across the lifespan. Exposure declined monotonically for ages older than 30 years, and a relatively higher frequency of PMP use than other forms of exposure in the younger groups. Participants who attended concerts tended also to frequent clubs, whereas PMP use was related only modestly to the other forms of exposure. These patterns correspond with what might be expected intuitively. However, each form of exposure had a similar relationship with tinnitus. Workplace noise exposure had a somewhat stronger influence on tinnitus frequency among older groups, perhaps reflecting greater exposure in those groups.

Some older participants in this study would have had considerable exposure to amplified music across their entire adult life, as phonograms have been widely available since the 1930s, and the first PMPs (portable cassette players) were widely available from the late 1970s. However, because PMPs have grown in popularity in more recent years, especially in younger people, patterns and duration of PMP exposure across the ages did vary substantially more than did concert and club attendance.

### 3.7 Tinnitus in younger people

Tinnitus has been found to be more common in those with audiometric hearing loss than in those without (Langguth et al., 2013, Shargorodsky et al., 2010) and, at least to some extent, to match the audiometric frequencies at which threshold elevations were seen (Norena et al., 2002). These observations provide evidence for a link between tinnitus and hearing loss. However, examination of quantitative data linking tinnitus to noise exposure in these population studies (Langguth et al., 2013, Shargorodsky et al., 2010) suggests that the linkages are often modest. Odds ratios are generally less than 2, and confusing patterns of results have been observed, for example that ‘frequent’ tinnitus has smaller, multivariate-adjusted ORs than ‘any’ tinnitus in association with leisure noise exposure (Shargorodsky et al., 2010).

Data reported here showed that, while poor SRTs were significantly associated with tinnitus, the effect size was small. We know from previous research using balanced sampling and even more participants than here (Moore et al., 2014) that there is a major discrepancy between the increase in SRT and self-reported hearing difficulty with increasing age. While data on these measures generally correlate (Thodi et al., 2013), we can only speculate that either respondents are reporting some aspect of their hearing that is not captured by either an audiogram or a speech-in-noise test, and/or that people's self-percept of their hearing difficulty is very variable. Self-reported hearing difficulty was more strongly associated with tinnitus but most people reporting ‘Usual’ or ‘Constant’ tinnitus (76%) still reported little hearing difficulty. These observations may be linked to the age of the sample in this study, who were mainly younger people with little reported hearing difficulty overall. Just 8% of the respondents who were not hearing device users reported anything greater than ‘Slight’ hearing difficulty while 29% of the sample reported ‘Sometimes’, ‘Usually’ or ‘Constantly’ experiencing tinnitus. Together, these results suggest that tinnitus occurs much more widely than does hearing loss, especially among younger adults. It is possible that the tinnitus experienced by younger people is of a different type, unconnected with hearing loss. However, we found little difference across age either in the moderate correlation between tinnitus and hearing difficulty or in the proportion of those with no hearing difficulty experiencing tinnitus occasionally or often (40–50%). It is thus possible that previous studies may have exaggerated the relationship between hearing loss and tinnitus in the general population. Further population research involving quantitative measurement of both speech-in-noise and tone detection will help to clarify the relationship with tinnitus.

### 3.8 Methodological considerations

To our knowledge, this is the first widescale epidemiological study incorporating a validated test of hearing that has been conducted entirely via the internet. This form of experiment has many advantages including efficiency, speed, number of participants and automated data collection. However, it does also include some disadvantages, the most obvious of which is biased sampling, as seen here for the age distribution and, presumably, socioeconomic factors including access to and knowledge of digital media and the form of publicity given to the study. Because of the large number of participants, it was possible to study age dependence up to the oldest decade, 65–75 years, beyond which range the number of responders fell below 50. Nevertheless, statistical power was compromised in some respects throughout the sample, for example in the relatively low number of users of hearing devices. On the other hand, the unintentional age bias towards younger people did provide more power to address the distinct nature of hearing in younger adults that we had not anticipated at the outset of the study.

Gender differences were also apparent, with fewer female participants among the younger age groups. Fortunately, these were also the groups where numbers were buoyant for both sexes, so we do not have a particular concern in this study. Different numbers and patterns of internet use between the sexes have been reported (Jones et al., 2009), with males tending to be more frequent users and more liable to visit ‘information’ sites such as the one used here. Among the major institutions (MRC, CCHMC) providing a large portion of the sample in this study, many employees are young, almost all are well educated, and the gender balance is weighted towards women.

Another methodological concern is suggested by the relatively high prevalence of tinnitus in this sample (29%) compared with, for example, UK Biobank (10–25% (Dawes et al., 2014, McCormack et al., 2014b)). This difference is magnified by the finding in all studies that tinnitus prevalence increases with age. The age range for the UK Biobank study was 40–69 years, with a mean age (58 years) well above that of this study. On the other hand, the questions asked in each study were somewhat different, so the observed difference in prevalence may not be robust for that reason. It is also possible that those with concerns about their hearing would be more inclined to participate in a study on the internet, perhaps with the expectation of gaining further insight into their difficulty. If that were the case, however, it is surprising that the proportion of participants in this study reporting tinnitus was so much higher than those reporting hearing difficulty, unlike UK Biobank.

### 3.9 Recent exposure to music or noise

When long-term workplace noise and music exposure were excluded, recent exposure to noise or music was associated with a significant increase in tinnitus reports and there was an age-related interaction. We also observed an association between recent and lifetime exposure. There are several reports of a high prevalence of temporary tinnitus following acute exposure to sounds, including music (Gilles et al., 2013, Keppler et al., 2015), but the quantitative relationship between recent or acute noise exposure and tinnitus is not well known. The longer-term effects of acute sound exposure have recently assumed increased importance with findings of inner hair cell ‘synaptopathy’ in animals exposed to levels of sound that do not appear to increase audiometric thresholds (Kujawa and Liberman, 2009). In humans, there is evidence of possible synaptopathy or neural gain changes in young adults reporting tinnitus, but with normal audiometry (Brotherton et al., 2015, Schaette and McAlpine, 2011). It is therefore possible that acute exposure to leisure music might have deleterious results for tinnitus. However, a recent animal study found only a limited relationship between synaptopathy and a common behavioral test of tinnitus in animals (Hickox and Liberman, 2014).

## Figures and Tables

**Table 1:** Tinnitus frequency across the total sample. Number and percentage of participants choosing each response to the Tinnitus question (Appendix B, Q5).

Tinnitus	Never	Rarely	Sometimes	Usual	Constant
Number	2044	1469	661	316	460
Percentage	41.3	29.7	13.4	6.4	9.3

**Table 2:** Influences on tinnitus. Summary of final multinomial regression model selected by likelihood ratio tests and AIC comparisons. **A**. Model coefficients. The first two rows show the odds of ‘Often’ and ‘Occasional’ tinnitus relative to no tinnitus (‘Never’). Other rows show odds ratios (ORs) for ‘Often’ and ‘Occasional’ tinnitus as a function of Age, Gender, Workplace Noise, overall Music Exposure (linear and quadratic terms) and the Age:Gender interaction. All parameters are relative to a participant with ‘baseline’ characteristics (mean age = 37.7, mean music exposure = 2.66, male, no workplace noise exposure). See Appendix C for further description and examples. **B**. Analysis of Deviance table and AIC.

A. Regression model: Tinnitus ∼ Age + Gender + Age:Gender + Noise + Music + Musicˆ2.				
	Odds	2.5% CI	97.5% CI	p
Often	0.315	0.272	0.365	<0.001
Occasional	0.939	0.845	1.044	0.243
	OR			
Often-Age	1.039	1.030	1.047	<0.001
Occasional-Age	1.001	0.995	1.007	0.786
Often-Gender(Female)	0.985	0.821	1.181	0.871
Occasional-Gender(Female)	1.153	1.015	1.310	0.028
Often-Noise(<1yr)	0.998	0.776	1.283	0.988
Occasional-Noise(<1yr)	1.322	1.111	1.573	0.002
Often-Noise(1–5yrs)	2.045	1.483	2.819	<0.001
Occasional-Noise(1–5yrs)	1.694	1.298	2.209	<0.001
Often-Noise(>5yrs)	2.602	1.800	3.761	<0.001
Occasional-Noise(>5yrs)	1.774	1.261	2.497	0.001
Often-Music	1.387	1.238	1.554	<0.001
Occasional-Music	1.349	1.241	1.468	<0.001
Often-Musicˆ2	1.048	0.975	1.125	0.202
Occasional-Musicˆ2	0.945	0.892	1.002	0.060
Often-Age:Gender(Female)	0.980	0.968	0.992	<0.001
Occasional-Age:Gender(Female)	0.984	0.975	0.993	<0.001

**B** Deviance table			
	Deviance	df	p
Age	111.6	2	<0.001
Gender	8.5	2	0.015
Age:Gender	17.4	2	<0.001
Noise	57.0	6	<0.001
Music	69.8	2	<0.001
Musicˆ2	9.2	2	0.01
Full model	AIC = 9793.3		

**Table 3:** Correlations between exposure types.

	Pearson correlation	Partial correlation controlling for Age	Correlation for participants <36 yrs
Clubs - Concerts	0.513	0.510	0.520
Clubs - PMP	0.289	0.173	0.125
Concerts - PMP	0.194	0.181	0.121

**Table 4:** Effect of each form of leisure music on tinnitus. Summary of multinomial regressions showing Deviance values and AIC for significant variables. Note that ‘Music’ here designates only the specific form (e.g. Concerts) of music exposure in each sub-table (**A** – **C**). Same model as Table 2.

	Deviance	df	p
**A** Pubs and Clubs			
Age	134.9	2	<0.001
Gender	7.8	2	0.021
Age:Gender	18.7	2	<0.001
Noise	62.4	6	<0.001
Music	38.2	2	<0.001
Musicˆ2	7.6	2	0.022
Full model	AIC = 9826.5		
**B** Concerts			
Age	132.6	2	<0.001
Gender	8.7	2	0.013
Age:Gender	20.1	2	<0.001
Noise	65.2	6	<0.001
Music	38.2	2	<0.001
Musicˆ2	5.0	2	0.081
Full model	AIC = 9829.1		
**C** Personal music players (PMP)			
Age	82.4	2	<0.001
Gender	11.0	2	0.004
Age:Gender	16.5	2	<0.001
Noise	72.8	6	<0.001
Music	44.0	2	<0.001
Musicˆ2	3.0	2	0.224
Full model	AIC = 9825.3		

**Table 5:** Modelling of speech reception threshold (SRT) by multiple linear regression. A. Final model selected by stepwise regression. ‘Music’ here is the composite measure. ‘Device’ denotes the sound delivery system (headphones, earbuds, etc). B. Simplified model containing only age, tinnitus and their interaction.

	Type-II SS	df	F	p
A Model: SRT ∼ Age + Ageˆ2 + Tinnitus*(Age + Noise + Recent Noise) + Age*(Music + Device) + Noise*Device + Musicˆ2.				
Age	4203	1	430.28	<0.001
Ageˆ2	871	1	89.19	<0.001
Tinnitus	194	2	9.95	<0.001
Noise	292	3	9.96	<0.001
Headphone	165	3	5.64	<0.001
Recent noise	57	3	1.93	0.122
Music	1	1	0.10	0.747
Musicˆ2	32	1	3.24	0.072
Tinnitus:Noise	202	6	3.44	0.002
Tinnitus:Age	108	2	5.54	0.004
Tinnitus:Recent noise	193	6	3.30	0.003
Age:Device	91	3	3.11	0.025
Age:Music	73	1	7.43	0.006
Noise:Device	267	9	3.0345	0.001
Residuals	47929	4907		
B Model: SRT ∼ Age + Ageˆ2 + Tinnitus*Age				
Age	5598	1	560.56	<0.001
Ageˆ2	859	1	86.05	<0.001
Tinnitus	292	2	14.63	<0.001
Tinnitus:Age	163	2	8.16	<0.001
Residuals	49361	4943		

**Table 6:** Hearing Difficulty. **A**. Numbers of participants reporting each frequency of tinnitus and each level of difficulty. **B**. Multinomial regression model summary, showing dominant influence of age and tinnitus.

A					
Tinnitus: Difficulty:	Never	Rarely	Sometimes	Usual	Constant
None	1229	811	276	67	85
Slight	740	578	318	182	259
Moderate	73	76	60	64	104
Great	2	4	7	3	12
Deaf	0	0	0	0	0

B Model: Hearing difficulty ∼ Noise + Recent noise + Music + Tinnitus			
	Deviance	d.f.	p
Age	248.6	2	<0.001
Noise	19.1	6	0.004
Music (total)	24.0	2	<0.001
Recent Noise	25.4	6	<0.001
Tinnitus	394.5	4	<0.001
Full model AIC = 8293.2			

**Table 7:** Effect of recent noise in a multinomial regression model that excluded workplace noise and music exposure Model: Tinnitus ∼ Age + Gender + Noise + Age:Gender + Age:Recent noise.

	Deviance	df	p
Age	130.1	2	<0.001
Gender	9.4	2	0.009
Recent noise	16.7	6	0.011
Age:Gender	23.5	2	<0.001
Age:Recent noise	13.9	6	0.031

## Appendix A Questions asked concerning music exposure in each decade of life

1. Concerts: How often would you say you went to gigs, concerts, and festivals?
  1 None
  2 Up to about 3 per year
  3 about once every other month
  4 about once a month
  5 about once a fortnight
  6 about once a week
  7 more than once a week
2. Clubs: How often would you say you went to loud clubs and discos?
  1 None
  2 Up to about 3 per year
  3 about once every other month
  4 about once a month
  5 about once a fortnight
  6 about once a week
  7 more than once a week
3. PMP: How often would you say you used any personal music player with headphones (e.g. Walkman, CD, MP3)?
  1 None
  2 Up to about 3 per year
  3 about once every other month
  4 about once a month
  5 about once a fortnight
  6 about once a week
  7 more than once a week

## Appendix B Questions asked concerning hearing, tinnitus, hearing device use, noise exposure, and recent exposure

1. Difficulty: Do you currently have any difficulty with your hearing?
  1 No difficulty
  2 Slight difficulty
  3 Moderate Difficulty
  4 Great Difficulty
  5 Cannot hear at all
2. Handicap: How often nowadays does any hearing difficulty restrict what you do socially or personally
  1 Never
  2 Rarely
  3 Sometimes
  4 Usually
  5 Constantly
3. Annoyance: How often nowadays does any hearing difficulty annoy or upset you?
  1 Never
  2 Rarely
  3 Sometimes
  4 Usually
  5 Constantly
4. Type-of-device: Do you currently use a hearing-aid or cochlear implant?
  1 Neither
  2 hearing aid (sometimes)
  3 hearing aids (most of the time)
  4 hearing aids (almost always)
  5 cochlear implants
  6 both a cochlear implant and a hearing aid
5. Tinnitus: How often nowadays do you get tinnitus (noises such as ringing or buzzing in your heard or ears) that lasts for more than 5 min?
  1 Never
  2 Rarely
  3 Sometimes
  4 Usually
  5 Constantly
6. Noise exposure: Have you ever worked in a place that was so noisy you had to shout to be heard?
  1 No, never
  2 Yes, less than 1 year
  3 Yes, for 1–5 years
  4 Yes, for over 5 years
7. Recent exposure: Have you had any loud noise or loud music exposure in the last 24 h?
  1 No, never
  2 Yes - noise
  3 Yes - music
  4 Yes - music and noise
8. Headphones: How do you plan to listen to the test?
  1 Built-in loudspeakers
  2 Separate loudspeakeres
  3 Earbuds
  4 headphones

## Appendix C Detailed interpretation of Table 2A

We explain here the meaning of some of the parameters in more detail and provide examples by reference to Table 2A. *Constant* describes the odds O_CN_(P_0_) = p(Constant)/p(Never) of having constant tinnitus for a baseline participant P_0_ (age = 37.7, music exposure = 2.66, male, no workplace noise exposure). For such a person, the probability of constant tinnitus is reduced to 0.315 times the probability of having no tinnitus. *Constant-Age* is the ratio of the constant-never odds for a participant who is one year older than baseline (but otherwise unchanged) to the baseline odds: O_CN_(P_0_ + 1 year)/O_CN_(P_0_). Its value of 1.039 (p < 0.001) shows that increasing age leads to higher odds of constant tinnitus for a baseline, male participant. Female participants showed a different trend (below). *Constant-Noise(* < *1yr)* is the constant-never odds ratio for less than 1 year workplace noise exposure relative to baseline: O_CN_(P_0_ with <1 occ. noise)/O_CN_(P_0_); its value 0.998 (p > 0.05) indicates that there is no evidence for a change in odds. *Const-Age:Gender(Female)* is the OR for a one-year age increase of female participants relative to a female baseline participant: O_CN_(S_0_ female + 1 year)/O_CN_(P_0_ female). The value 0.980 (p < 0.001) indicates odds *decreasing* with age for female participants.
